# Effect of milling on natural radioactivity of Egyptian zircon sand: an assessment of alpha and beta radiation emissions

**DOI:** 10.1007/s10653-026-03350-1

**Published:** 2026-07-15

**Authors:** Kolthoum I. Othman, Ola Makld, Mahmoud A. Mosleh, M. El-Sayed Ali, S. El-Houte

**Affiliations:** Metallurgy Department, Nuclear Research Centre (NRC), Egyptian Atomic Energy Authority, Cairo, Egypt

**Keywords:** Zircon sand, Planetary milling, Natural radioactivity, Alpha and beta activity concentrations

## Abstract

This study aims to investigate the effect of milling on the natural radioactivity of Egyptian zircon sand, focusing on the gross alpha (α) and goss beta (β) activity concentrations, with additional evaluation of calcination and sulfuric acid leaching. Zircon samples were milled for different durations, and their gross α and gross β activity concentrations were measured using an ATOMTEX AT1329 sample counter. The measurements showed that both gross α and gross β apparent activity concentrations increased with milling time, reaching maximum values of 3.5 ± 1.4 Bq/g and 7.2 ± 2.2 Bq/g, respectively, in the sample milled for 36 h. These values increased to 7.7 ± 3.1 Bq/g and 14.72 ± 4.4 Bq/g after calcination, while acid leaching resulted in a pronounced decrease in these values to 4.5 ± 1.7 Bq/g for alpha and 9.6 ± 2.9 Bq/g for beta. Milling reduces particle size and increases the escape probability of α and β particles from the sample bulk, while calcination removes the polyamide layer formed during milling, which acts as a barrier to particle detection. These findings highlight the potential radiation hazards associated with zircon sand processing to safeguard occupational health and environmental safety in industries working with this material.

## Introduction

Zircon (ZrSiO_4_) is a heavy mineral found in a variety of rocks and in abundant deposits worldwide. It possesses several properties that make it an important industrial material. It is highly stable at elevated temperatures and exhibits excellent characteristics, including high resistance to thermal shock, low thermal conductivity, and chemical inertness. In addition, zircon is non-magnetic, electrically non-conductive, and has favourable optical properties (Arnold, 2022; IAEA, 2007; Mohammed et al., 2024). Because of these characteristics, zircon is widely used in the ceramics, refractories, abrasives, foundry, and opacifier industries (IAEA, 2007; Mohammed et al., 2024; Samin et al., 2020). Additionally, it is an ancient mineral that preserves geological information from the formation of the Earth to the present day, making it valuable for radiometric dating and geochronology. Furthermore, its wide colour range and high refractive index make it a popular gemstone in the jewellery industry (Arnold, 2022). Owing to these unique properties, zircon and its derivatives have gained significant attention as strategic industrial materials.

Zircon sand serves as a raw material for producing valuable ceramic compounds used in various technical fields. It is an important source of zirconium and hafnium, both of which are essential for industrial applications and scientific research (Arnold, 2022). Zircon is also a primary feedstock for the synthesis of other zirconium-based materials (Arnold, 2022; Lubbe & Crouse, 2023). Most importantly, zirconium oxide (zirconia) is a key high-performance base material, for advanced technical and functional ceramics. Zirconia ceramics are used in the nuclear industry for manufacturing critical components, such as crucibles for melting platinum and rare earth elements, and in the continuous casting of steel. They are also employed in sensors, high-temperature structural components (Fedorov & Yarotskaya, 2021), solid oxide fuel cells (Yamamoto et al., 1998; Zakaria et al., 2020), dentistry (Thakare, 2012; Volpato et al., 2011), and in many other advanced applications (Chitoria et al., 2023; Stefan et al., 2023; Szabó et al., 2022; Yang et al., 2024).

Zircon sand contains significant concentrations of naturally occurring radioactive materials (NORM), mainly thorium-232 (^232^Th) and uranium-238 (^238^U), which substitute for zirconium in the crystal lattice during mineral formation (Hollitt et al., 1995; IAEA, 2007). These radionuclides undergo radioactive decay over time, eventually producing lead (Pb) as a stable end-product (Arnold, 2022; IAEA, 2007; van Es, 2008). By knowing the rate at which this decay occurs, scientists can measure trace amounts of these elements in zircon crystals and determine the age of the rocks in which they formed (Arnold, 2022).

In Egypt, zircon is found among the components of black sand along the Mediterranean coast, extending from east of Rosetta to Al-Arish for approximately 400 km. In addition to zircon, Egypt’s black sand deposits contain substantial reserves of other strategic and economically important heavy minerals, including ilmenite, garnet, magnetite, rutile, and monazite. The radioactivity of the black sands is mainly attributed to uranium and thorium series mostly related to monazite, zircon, garnet and ilmenite. Zircon contains smaller but measurable amounts of uranium (0.04–0.11%) and thorium (0.02–1.19%), whereas monazite is recognized as the principal carrier of natural radioactivity because of its high thorium content (6.05 wt.%) and, to a lesser extent, uranium content (0.06 wt.%) (Dabbour, 1995; El-Kammar et al., 2011). The variation in uranium and thorium levels in zircon is due to the presence of several varieties of beach zircon, whereas they remain relatively constant in monazite because it belongs to almost one variety (Dabbour, 1995). Ore dressing for the concentration and separation of zircon and other heavy minerals from black sand have been covered in previous publications (Abd El_Kareem, 2019; Abdel-Rehim, 2005; Diab et al., 2022; EL-Afandy et al., 2015; Fawzy et al., 2024; Rabee et al., 2022).

The Egyptian Nuclear Materials Authority (ENMA) has also established a plant for the industrial-scale exploitation of Egyptian placer deposits in the Rosetta area to extract and separate the economic heavy minerals concentrates of zircon, monazite, and rutile (Diab et al., 2022; EL-Afandy et al., 2015). Zircon sand is separated from associated heavy minerals and light minerals, such as quartz and clay, by means of mechanical, physical and chemical treatment to obtain purified zircon (EL-Afandy et al., 2015; El-Kammar et al., 2011). The extracted zircon is classified according to its uranium and thorium content. Colourless, water-clear zircon which represents the purest variety and contains the lowest concentrations of uranium and thorium, with average values of approximately 0.046% and 0.020%, respectively, making it suitable for ceramic applications. In contrast, zircon varieties enriched in radioactive elements are more suitable for the extraction of nuclear raw materials (Dabbour, 1995). Zircon sand used in the present work, therefore, belongs to the category suitable for ceramic applications because of its relatively low radioactive element content.

For many ceramic and industrial applications, zircon sand must first be milled to smaller particle sizes because its natural particle size of 100–200 μm is considered too coarse for effective processing (IAEA, 2007). The production of fine zircon powders through milling enhances powder reactivity by decreasing particle size and consequently increasing the specific surface area (Hollitt et al., 1995; Mohammed et al., 2024). Mechanical comminution can be achieved through impact, attrition, shear (grinding), and compression (hammering) methods. The general phenomena observed during solid-state size reduction are governed by fracture mechanics, including crack nucleation, propagation, and eventual fracture, which leads to the generation of new surfaces (Baláž, 2008).

The kinetic energy in the milled aggregate is partially transformed into mechanical stresses within the material, causing disintegration. Achieving smaller particle sizes requires the application of greater shear stress to each particle to promote further fracture. Fine milling can be carried out using devices such as planetary ball mills, centrifugal mills, and attritor mills. The planetary ball mill stands as the most widely utilized device for laboratory-scale ultrafine grinding. It is favored because the milling balls can attain impact energies up to 40 times greater than those produced by gravitational acceleration. Therefore, planetary ball milling is well suited for high-speed, high-energy milling of small quantities of precursor powders (Baláž, 2008; Burmeister & Kwade, 2013).

Owing to the presence of trace levels of naturally occurring radionuclides in zircon sand, careful consideration is needed regarding the structural changes that occur during milling. Zircon milling does not produce material with radionuclide activity concentrations higher than those of the original zircon sand feedstock (IAEA, 2007). During the milling, particle size reduction and the associated increase in surface area may enhance zircon grains exposure, which can lead to measurable changes in radiological counts. Understanding these effects is important for the safe handling and processing of zircon-based raw materials.

The objective of this study is to evaluate the effect of milling on the measured alpha and beta radioactivity of zircon sand. In this context, pure zircon sand samples supplied by ENMA were subjected to controlled high-energy planetary milling for different durations. The variations in gross alpha and gross beta activity concentrations before and after milling were examined. In addition, the effects of calcination and acid leaching on the milled zircon were studied in relation to their influence on these activity concentrations.

## Experimental work

The starting material used in this study was high-grade Egyptian zircon sand (ZrSiO_4_, 98%) extracted from the black sand deposits located in the Rosetta region of Egypt. The material was supplied by the Egyptian Nuclear Materials Authority (ENMA) after undergoing physical and chemical beneficiation. The zircon samples were dry-milled in a planetary mill (Retsch PM400-type, Fritsch) for various milling durations: 2, 12, 24, 36, and 48 h. Polyamide bowls of 250 mL capacity and zirconia balls were used as the milling medium. The milling speed was maintained at 200 rpm, and the process was paused for 5 min every 30 min to allow the system to cool. Both milled and un-milled zircon powders were calcined in air at 900 °C for 2 h in a carbolite furnace (GPC 1300, UK). The calcined powders were then leached with sulfuric acid by mixing the zircon sand with the H_2_SO_4_ acid solution and agitating the mixture to enhance the leaching process.

Zircon powders were characterized as follows: thermal analysis was performed using a thermo-gravimetric analyzer (TGA-50, Shimadzu) at a heating rate of 10 °C/min. Chemical composition was determined by X-ray fluorescence (XRF; SPECTRO Analytical Instruments GmbH). The crystal structure was examined using an X-ray diffractometer (XRD3A, Shimadzu) and quantitative phase analysis was carried out using X’Pert High Score software. Crystallite size was calculated from X-ray diffraction peak broadening using Scherrer’s equation. The Brunauer–Emmett–Teller (BET) surface area was measured using a gas adsorption analyzer (BEL SORP MAX).

The gross alpha and beta activity concentrations were measured using an ATOMTEX AT1329 instrument equipped with a Phoswich detector: a plastic scintillator for beta particles and ZnS(Ag) for alpha particles. The instrument was calibrated to determine the counting efficiency for each radiation type. For beta calibration, 1.0 g of analytical-grade potassium chloride (KCl) was finely ground and uniformly spread over a 4 cm^2^ planchet. The known specific activity of ^40^K in KCl (16.3 ± 1 Bq/g) was used as the reference activity. The KCl standard and the samples were measured under identical geometric conditions. For alpha calibration, a sealed Americium-241 (^241^Am) source (0.7 µCi, 25.9 ± 5 kBq) covered with 1 µm gold film was used as a reference point source. Measurements were performed at the same source-to-detector distance and counting geometry. All samples were prepared with the same mass and geometry to minimize self-absorption effects and ensure comparability across the dataset. The gross alpha and gross beta activity concentrations of the samples were determined using the net count rate obtained after background subtraction. The net count rate, ${C}_{net}$, was calculated as:

1 $${C}_{net}= {C}_{gross}- {C}_{bg}$$

where ${C}_{gross}$​ is the gross count rate of the sample and ${C}_{bg}$​ is the background count rate.

$${C}_{gross}=\frac{{N}_{gross} }{t},{ C}_{bg}=\frac{{N}_{bg} }{{t}_{bg}}$$

where ${N}_{gross}$​ and ${N}_{bg}$​ are the gross and background counts, respectively, while t and ${t}_{bg}$​ are the corresponding counting times.

The counting efficiency, ε, for each radiation channel was determined using a standard material with known activity according to:

2 $$\varepsilon =\frac{{C}_{net,std} }{{A}_{std}}$$

where ${C}_{net,std}$​ is the net count rate of the standard and ${A}_{std}$​ is its known activity.

The activity concentration of the sample, ${A}_{sample}$​, was then calculated as:

3 $${A}_{sample}=\frac{{C}_{net,sample} }{\varepsilon }$$

where ${C}_{net,sample}$,​ is the net count rate of the sample.

The standard uncertainty associated with the net count rate was obtained by propagating the counting statistics of the gross and background measurements:

4 $$u\left({C}_{net}\right)= \sqrt{{u{(C}_{gross})}^{2}+{u{(C}_{bg})}^{2}}$$

The relative combined uncertainty in the sample activity concentration was calculated using the law of propagation of uncertainty:

5 $$\frac{u({A}_{sample})}{{A}_{sample}}= \sqrt{{\left(\frac{u({C}_{net,sample})}{{C}_{net,sample}}\right)}^{2}+{\left(\frac{u(\varepsilon )}{\varepsilon }\right)}^{2}}$$

and

$$\frac{u({C}_{net,std})}{{C}_{net,std}}= \frac{u(\varepsilon )}{\varepsilon }$$

Hence

6 $${\left(\frac{u({A}_{sample})}{{A}_{sample}}\right)}^{2}= {\left(\frac{u({C}_{net,sample})}{{C}_{net,sample}}\right)}^{2}+{\left(\frac{u({C}_{net,std})}{{C}_{net,std}}\right)}^{2}$$

The resulting combined standard uncertainties were approximately 40% for alpha activity and 30% for beta activity, with higher uncertainties observed for alpha measurements due to lower counting efficiency and greater sensitivity to self-absorption effects.

### Limitation regarding sample matrix

It is important to note that the calibration standards used in this study (KCl and a sealed ^241^Am source) do not possess the same matrix characteristics as the zircon sand samples. Self-absorption of radiation, particularly alpha particles, depends strongly on sample density, composition, particle packing, thickness, and geometry (Burns, 1982; Gilmore & Joss, 2024; Vargas & Timón, 2005). The ^241^Am source is a thin sealed source with negligible self-absorption, whereas the sand samples exhibit non-negligible self-absorption effects. Similarly, the density and effective atomic number of KCl differ from those of zircon matrix. Therefore, the detection efficiencies derived from these calibration standards may not exactly represent the true counting efficiency of the zircon samples. Since no explicit self-absorption correction factor was applied, the reported alpha and beta activity concentrations should be regarded as apparent rather than absolute values. The reported values are therefore likely to represent a conservative underestimate of the true activity, particularly for gross alpha measurements. Accordingly, the adopted methodology is intended primarily for comparative evaluation and screening of the milling effect under identical measurement conditions, rather than for high-accuracy absolute radioactivity determination.

## Results

### Characterization of the as-received Rosetta zircon sand

**Fig. 1:**
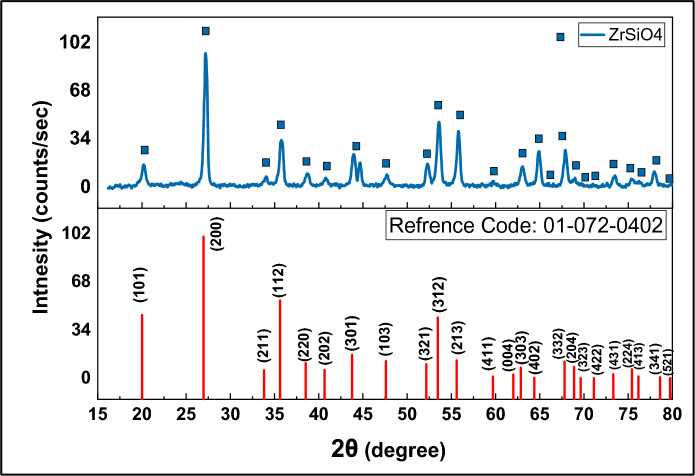
X-ray diffraction pattern of as-received zircon sand

The XRF chemical analysis of the as-received zircon, in wt.%, is presented in Table 1 (El-Kammar et al., 2011). The analysis shows that the major constituents are zirconia, containing approximately 1% hafnia and silica in a 2:1 weight ratio. Minor quantities of radioactive elements, 300 ppm of U_3_O_8_ and 100 ppm of ThO_2_, are also identified. Based on the molecular weights of these oxides, the raw zircon sand contains only 254.4 mg/kg of U and 87.9 mg/kg of Th. These results confirm the high purity grad of zircon sand used in this study. XRD patterns of the as-received zircon (Fig. 1) reveal that all diffraction peaks correspond to the ZrSiO_4_ phase and match well the Miller indices listed in JCPDS No. 01-072-0402.​ No other phases were detected in the XRD patterns.

**Table 1:** XRF analysis of as-received zircon sand

Element	wt.%	Element	wt.%	Element	wt.%
ZrO_2_	66.40	La_2_O_3_	0.01	P_2_O_5_	< 0.01
SiO_2_	31.83	Nd_2_O_3_	< 0.01	Pr_2_O_3_	< 0.01
HfO_2_	1.44	Gd_2_O_3_	< 0.01	Sm_2_O_3_	< 0.01
U_3_O_8_	0.03	Ho_2_O_3_	< 0.01	Dy_2_O_3_	< 0.01
Y_2_O_3_	0.03	Tm_2_O_3_	< 0.01	Er_2_O_3_	< 0.01
ThO_2_	0.01	CaO	< 0.01	Yb_2_O_3_	< 0.01
Ce_2_O	0.01	Fe_2_O_3_	< 0.01	PbO	< 0.01

### Milling of zircon sand

**Fig. 2:**
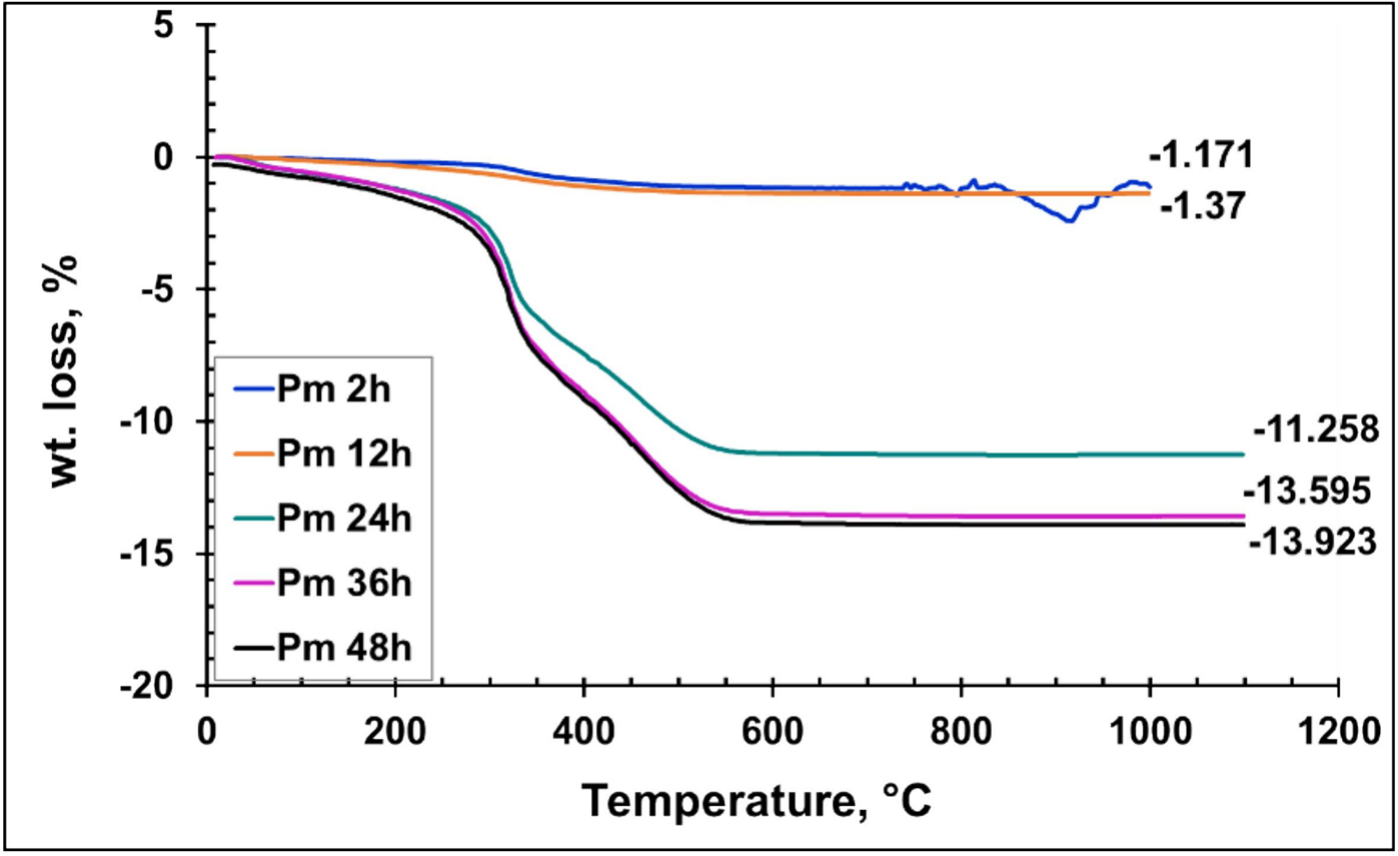
Thermogravimetric analysis (TGA) of zircon sand powders milled for different durations

Figure 2 presents the thermogravimetric analysis (TGA) of the milled zircon powders. Weight loss increased with prolonged milling time, with a significant loss observed after 24 h of milling, whereas milling for up to 12 h resulted in only minor weight loss. This weight loss is likely due to the burning of the successive polyamide layers built up on the surface of the zircon powder during extended milling.

**Fig. 3:**
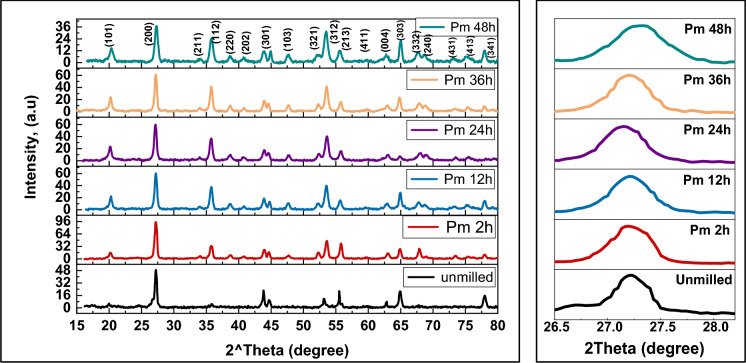
X-ray diffraction patterns of zircon powders milled for various durations

**Fig. 4:**
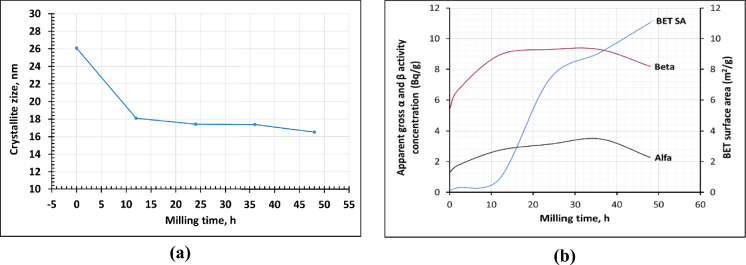
**a** Crystallite size as a function of milling time, and **b** variation in specific surface area, and apparent gross alpha and gross beta activity concentrations with milling time

The XRD patterns of zircon sand powders milled for various durations (Fig. 3) exhibit moderate peak broadening with increasing milling time. It is interestingly observed that peaks at 2θ values of 20°, 35.6°, and 53.3° [corresponding to the (101), (112), and (312) planes, respectively] show an increase in intensity with milling time. Meanwhile, peaks at 2θ values of 33.8°, 38.5°, 40.7°, 47.6°, 52°, 67.8°, and 73.3° [corresponding to the (211), (220), (202), (103), (321), (332), and (431) planes, respectively] are absent in the un-milled powder but become clearly visible in the milled samples. In contrast, the peaks observed in the un-milled powder at 2θ values of 64.5° and 78° [corresponding to the (303) and (341) planes] show a marked decrease in intensity or disappear in the milled powders. These observations may be attributed to the formation of preferred orientation (texturing) in some crystallographic planes as a result of severe plastic deformation during milling (Sato et al., 2022; Sübütay & Şavklıyıldız, 2023). To further examine the structural changes induced by milling, crystallite size as a function of milling time is presented in Fig. 4a. The results show that the average crystallite size decreases sharply from 26 to 18 nm as milling time increases up to 12 h, after which only a marginal further decrease is observed, reaching a minimum value of 16.5 nm at 48 h.

Figure 4b illustrates the variation in specific surface area, as well as apparent gross alpha and gross beta activity concentrations with respect to milling time. A sharp increase in BET-specific surface area is observed as milling time increases from 2 to 48 h, indicating a reduction in particle size. It can also be seen that the apparent activity concentrations of both gross alpha and gross beta increase with milling time, except for the sample milled for 48 h, where a decrease is observed. In addition, the gross beta activity concentration showed higher values compared to gross alpha activity concentration.

### Effects of different treatments on zircon sand

**Fig. 5:**
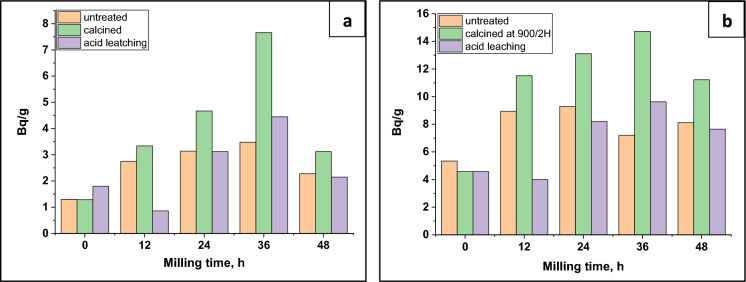
Apparent activity concentrations of **a** gross alpha, and **b** gross beta in milled zircon before and after different treatments

As previously mentioned in the experimental section, the milled and un-milled zircon sand samples were calcined at 900 °C for 2 h, and the calcined samples were subsequently leached with sulfuric acid. The radioactivity measurements of these samples, taken before and after each treatment, are graphically represented in Fig. 5. The figure showed that both apparent gross alpha and gross beta activity concentration increased with milling time up to 36 h, reaching maximum values of 3.5 ± 1.4 Bq/g for α and 7.2 ± 2.2 Bq/g for β. After calcination, these apparent activity concentrations further increased to 7.7 ± 3.1 Bq/g and 14.7 ± 4.4 Bq/g. In contrast, subsequent sulfuric acid leaching significantly reduced the measured apparent activity concentrations to 4.5 ± 1.7 Bq/g for alpha and 9.6 ± 2.9 Bq/g for beta. The as-received Egyptian zircon sand exhibited apparent gross α and gross β activity concentrations of 1.3 ± 0.5 Bq/g and 5.4 ± 1.6 Bq/g, respectively. These results demonstrate the significant effects of milling, calcination and acid leaching processes on the measured apparent radioactivity of zircon sand.

## Discussion

Planetary milling is a mechanical process commonly used to reduce particle size and enhance material activity (Burmeister & Kwade, 2013). When applied to Egyptian zircon sand, this process leads to a reduction in particle size and an increase in surface area. The effect of high-energy milling on zircon sand involves a complex interplay of amorphization, recrystallization, agglomeration, and surface layer formation (Baláž, 2008). During milling, zircon sand interacts with zirconia balls within the polyamide bowl, initiating a series of mechanical effects that alter the material's consistency. The dynamics of abrasion and collision between the zircon particles and the zirconia balls within the polyamide environment result in various structural transformations—ranging from the disruption of crystalline order to the formation of new microstructures—while simultaneously balancing dispersion, aggregation, and polyamide layer formation.

One of the intriguing findings of this study on the high-energy milling of Egyptian zircon sand is the observed variation in measured gross α and gross β apparent activity concentrations with milling time (Fig. 4b). It should be emphasized that zircon milling does not generate additional radionuclides or increase the true activity concentration beyond that present in the original zircon feedstock (IAEA, 2007; Righi et al., 2005). In zircon, uranium and thorium atoms and their decay elements, are bound within the crystal structure through isomorphic substitution for a small fraction of zirconium sites (Hollitt et al., 1995; IAEA, 2007; Tyler & Minnitt, 2004). High-energy milling induces progressive structural disorder, particle size reduction, and an increase in specific surface area accompanied by improved powder homogeneity (Baláž, 2008), which can lead to measurable variations in the detected radiological counts.

At the initial stages of milling, particle size reduction and the consequent increase in surface area significantly enhance the exposure of zircon grains (Aral et al., 2007; Mohammed et al., 2024). This structural refinement shortens the average path length required for emitted particles to emerge from the matrix, reducing internal self-absorption, and allowing a greater fraction of alpha and beta particles to escape from the sample. This enhances the effective counting efficiency, yielding higher measured apparent activity concentrations without an actual change in isotopic content (Aral et al., 2007). In contrast, prolonged milling durations resulted in a decrease in the apparent gross alpha and gross beta activity concentrations. This decrease may be attributed to thermal and microstructural changes induced during extended milling, such as particle agglomeration and partial sintering, which alter particle size distribution and morphology (Baláž, 2008; Zhang et al., 2019), thereby intensifying self-absorption within the sample matrix (Verma et al., 2024). To verify this effect, the theoretical maximum activity concentrations of the as-received zircon sand were estimated as 27.3 Bq/g for gross alpha and 20.3 Bq/g for gross beta from the U and Th impurities identified by XRF. These values represent the upper limit in the absence of self-absorption and confirm that the un-milled sand is strongly attenuated by the matrix.

Excessive dry milling can produce extremely fine powders that tend to re-agglomerate due to their high surface energy and may behave like larger particles, while localized frictional heating may promote partial sintering of particles (Baláž, 2008). As a result, radionuclide-bearing surfaces may become embedded within larger secondary aggregates, increasing the effective material thickness through which emitted particles must travel before reaching the detector, thereby reducing the detection efficiency (Verma et al., 2024).

In addition, localized heating generated during prolonged high-energy milling can lead to the loss of certain decay products within the uranium series, particularly Polonium-210 (^210^Po) and Lead-210 (^210^Pb). ^210^Po, a highly volatile alpha emitter, may partially escape at localized frictional hotspots. While ^210^Pb, a beta emitter, requires significantly higher temperatures to volatilize. The loss of ^210^Pb is primarily driven by mechanical degradation, where intense milling causes the micro-flaking of brittle ^210^Pb-rich surface coatings into fine dust (Cook et al., 2018; Hofmann et al., 2000; IAEA, 2007). These combined thermal and mechanical effects may alter the measured alpha and beta activity concentrations in the ultrafine fractions.

The results also showed that, the gross beta apparent activity concentration was higher than that of alpha particles (Fig. 4). This difference is most likely related to the greater penetration range of beta particles in matter and their lower self-absorption compared with alpha particles. Because beta particles are lighter, they possess greater penetration capabilities, allowing them to escape the bulk material more freely. In contrast, alpha particles are massive, highly ionizing nuclei, they are strongly attenuated by the solid zircon framework and exhibit a highly restricted effective range (Verma et al., 2024).

Another noteworthy aspect of this study is the formation of a polyamide-derived contamination layer on the milled zircon powder. High-energy milling involves intense shear, impact, and frictional forces that can induce mechanical wear on the milling media, particularly when processing hard and abrasive materials like zircon sand (IAEA, 2007). While the zirconia grinding balls remain highly resistant, the softer polyamide bowls undergo minor degradation. Consequently, microscopic organic polymer fragments are scraped from the inner walls of the bowl and incorporated into the grinding matrix, and may resulting in the deposition of a thin polyamide coating on the surfaces of the milled zircon particles. This coating may attenuate the emitted alpha and beta particles, enhancing self-absorption within the sample matrix, thereby lowering the measured apparent activity concentrations.

Interestingly, a substantial increase in both alpha and beta apparent activity concentrations was observed after calcination, which can likely be attributed to the thermal removal of the organic polyamide coating. The elimination of this organic layer increases the relative fraction of the radioactive zircon matrix within the measured mass, leading to a higher activity concentration per unit mass. In addition, removal of this organic barrier may enhance the escape probability of the emitted radiation toward the detector, thereby increasing the measured count rates. This increase may also be explained by a reduction in the effective linear attenuation coefficient between the zircon particles and the radiation detector. Elimination of this coating layer restores a more direct transmission path for the emitted radiation, thereby reducing attenuation and enhancing detection efficiency (Al-Rawi, 2014). It should be noted that while this organic coating can be readily eliminated via calcination, utilizing metallic milling media introduces permanent impurities. When steel or hardened chromium-steel milling media, mechanical abrasion inevitably introduces trace amounts of iron, nickel, and chromium into the powder. Such metallic contamination may alter the elemental composition of the final product and potentially affect its final properties (Baláž, 2008; Burmeister & Kwade, 2013).

By contrast, subsequent sulfuric acid leaching of the calcined, milled zircon sand resulted in a significant reduction in the measured apparent activity concentrations, which may be attributed to the removal of surface-associated radionuclides. Acid leaching is a chemical treatment used to remove surface impurities and radionuclides primarily uranium, thorium, and their decay products, from zircon sand without significantly changing its structure (Afifi et al., 2018; Aral et al., 2007; Hollitt et al., 1995; Mohammed et al., 2024). Various acids can be employed in the leaching process, including hydrochloric acid, nitric acid, sulfuric acid, and certain strong organic acids (Hollitt et al., 1995). Sulfuric acid (H_2_SO_4_) is considered particularly effective for leaching uranium- and thorium-bearing species from zircon sand due to its high leaching efficiency, relatively low cost, and strong ability to dissolve associated impurities.

The high efficiency of H_2_SO_4_ leaching is attributed to its ability to attack and dissolve the particle surface matrix, thereby facilitating the removal of uranium and thorium species into the liquid leachate in the form of soluble sulphate complexes, namely [UO_2_(SO_4_)_2_]^2−^ and [Th(SO_4_)_3_]^2−^ (Mohammed et al., 2024). A Previous study (Afifi et al., 2018) reported that H_2_SO_4_ provides higher thorium extraction efficiency (91.95%) than hydrochloric (HCl), nitric (HNO_3_), and phosphoric (H_3_PO_4_) acids under comparable experimental conditions. However, H_2_SO_4_ leaching may not effectively remove radium nuclides, Radium-226 (^226^Ra), as dissolved chemical species. Due to the formation of insoluble radium sulfate (RaSO_4_), these nuclides may either remain adsorbed onto the grain surfaces or be physically removed as a precipitate during subsequent washing stages. Consequently, while uranium and thorium species are transferred into the liquid leachate, ^226^Ra remains structurally fixed and immobilized within the solid ceramic residue (Aral et al., 2007; Hollitt et al., 1995).

The results of the present study showed that the H_2_SO_4_ leaching efficiency significantly improved following milling (Fig. 5). This improvement may be attributed to the particle size reduction and increased surface area produced by milling, which could enhance the reactivity of the zircon surface toward H_2_SO_4_ leaching and facilitate the partial removal of surface-associated radionuclides and their decay products. These results are in a line with the findings of Aral et al. (Aral et al., 2007) who reported that grinding significantly improved U and Th removal and reduced the total activity in the leach products to below 70 Bq/g. They attributed this behavior to the increase in the surface-area-to-volume ratio caused by grinding. Ruan et al. (2019) also reported that reducing particle size increases the contact area with leaching agents, thereby accelerating the reaction rate and improving leaching efficiency. During grinding, mineral particles are subjected to impact, friction, and localized heating, which increase lattice stress and energy and thus lower the activation energy required for the leaching reaction (Ruan et al., 2019).

The acid dissolves the radioactive elements, transferring them into the liquid solution while leaving the zircon matrix largely unaffected (Mohammed et al., 2024). The solution containing dissolved radionuclides can then be treated to separate and remove the radioactive elements using techniques such as precipitation, ion exchange, or solvent extraction (Ma et al., 2023). For safe disposal, the extracted radionuclides may be stabilized by incorporating them into a solid matrix or another suitable immobilization medium (Khelurkar et al., 2015).

Overall, based on the aforementioned discussion, the observed variations in alpha and beta counts do not reflect a change in the intrinsic radioactivity of the zircon material, but are more likely due to changes in sample morphology, sample–detector interaction, and internal self-absorption, which affect the apparent activity concentrations.

## Conclusions

This study shows that high-energy planetary milling affects the apparent natural radioactivity of Egyptian zircon sand by reducing particle size and increasing the exposed surface area. The as-received zircon sand exhibited apparent gross α and β activity concentrations of 1.3 ± 0.5 Bq/g and 5.4 ± 1.6 Bq/g, respectively. With increasing milling time, both values increased and reached maximum levels of 3.5 ± 1.4 Bq/g for α and 7.2 ± 2.2 Bq/g for β after 36 h, mainly due to improved powder homogeneity, greater surface exposure, and reduced self-absorption. At longer milling times, the measured activity concentrations decreased, which may be related to particle agglomeration, localized heating, and changes in attenuation and counting efficiency. Calcination of the milled zircon further increased the apparent activity concentrations to 7.7 ± 3.1 Bq/g for α and 14.7 ± 4.4 Bq/g for β, mainly due to the removal of the polyamide coating layer formed during milling, which reduced attenuation and improved detection efficiency. In contrast, subsequent sulfuric acid leaching resulted in a significant reduction in the apparent α and β activity concentrations to 4.5 ± 1.8 Bq/g and 9.6 ± 2.9 Bq/g, respectively, indicating partial removal of surface-associated radionuclides and their decay products. Overall, the results show that milling, calcination, and acid leaching strongly influence the measured apparent radioactivity of zircon sand and should be carefully considered in process control, occupational safety, and environmental protection. Finally, the findings presented in this study are specific to the Egyptian Rosetta zircon sand (ZrSiO_4_, 98% purity) and the particular experimental procedures employed herein. These results are inherently limited to the specific milling, calcination, and leaching conditions investigated, reflecting variations in the measured radiological response arising from matrix and physical changes rather than a true loss of radioactive material.

## Data Availability

No datasets were generated or analysed during the current study.
